# Falls risk perception and its influencing factors in elderly patients with hip fracture: a cross-sectional study

**DOI:** 10.1186/s12877-026-08030-4

**Published:** 2026-07-25

**Authors:** YaoYao Du, Chunyan Wang, Rong Song, Meng Wu, Yaqing Zhang, Rong Hu

**Affiliations:** Department of Orthopedics, School of Medicine, Mianyang Central Hospital, University of Electronic Science and Technology of China, Mianyang, 621000 China

**Keywords:** Hip fracture, Falls, Risk perception, Older adults

## Abstract

**Objectives:**

To explore the status and influencing factors of fall risk perception in elderly hip fracture patients, and to inform clinical fall prevention strategies.

**Methods:**

This cross‑sectional study enrolled 162 elderly patients with hip fracture from Mianyang Central Hospital. Data were collected using a general information questionnaire, the Fall Risk Perception Questionnaire (FRPQ), Perceived Social Support Scale (PSSS), a simplified anxiety‑depression scale, and the Fall Efficacy Scale. One‑way ANOVA, Pearson correlation, and multiple linear regression were used for analysis.

**Results:**

Mean age was 78.33 ± 9.32 years, and mean FRPQ score was 35.43 ± 12.00 (out of 78). ANOVA showed significant associations with age, education, payment method, and first‑ambulation pain (*P* < 0.05); moderate‑to‑severe pain was linked to lower scores. Correlation analysis revealed negative correlations with anxiety (r= ‑ 0.358) and depression (r= ‑ 0.520), and positive correlations with social support (*r* = 0.444) and fall efficacy (*r* = 0.600) (*P* < 0.01). Regression identified fall efficacy, social support, and high school/technical secondary education as positive independent predictors, while anxiety, depression, and moderate‑to‑severe pain were negative predictors ( R²=0.395, *P* < 0.05).

**Conclusions:**

Postoperative fall risk perception is relatively low in this population. Enhancing fall efficacy, social support, and education, while addressing anxiety, depression, and pain, may improve risk perception and reduce falls. Clinical attention should target patients with mood disorders, severe pain, and low education level, with comprehensive interventions including analgesia, psychological support, and efficacy‑building training.

## Introduction

With the accelerating aging of the population, an increasing number of elderly patients are suffering from hip fracture (HF) [1, 2]. Hip fracture in the elderly is a very serious osteoporotic fracture characterised by high incidence, high disability rate and high mortality [2–4]. Compared with conservative treatment, surgery is the main treatment for elderly hip fracture patients, which can effectively reduce the risk of death and improve quality of life [3, 5–8]. However, elderly patients after hip fracture surgery often experience pain, infection, kinesiophobia and fear of falling. Under the influence of these factors, the risk of falling increases, which is not conducive to the implementation of early rehabilitation training [1, 2, 9]. Studies have shown that approximately 45% of elderly hip fracture patients are at risk of falling during the rehabilitation period [7]. Falls are another important factor leading to morbidity and mortality in the elderly. More than 35% of patients who fall will experience re-fracture, soft tissue injury, head trauma, psychological trauma or even death, seriously affecting their quality of life and health outcomes [10–12]. Research indicates that the key to fall prevention is fall risk assessment. In addition to objective risk assessment by healthcare professionals, the elderly patients’ own perception of their fall risk is also crucial [10, 12–15]. Falls risk perception in the elderly emphasises thinking about fall risk from the perspective of the risk subject, i.e., the elderly themselves, focuses on their comprehensive assessment and true feelings of their own fall risk, and highlights their initiative in self-management of falls, which can better predict activity behaviour and fall-related outcomes [14]. At present, clinical research on elderly hip fracture mainly focuses on early mobilisation, postoperative nursing, complications, fear of falling, etc. [1–3, 8, 9, 16–18]., while studies on the current status of falls risk perception after surgery are limited.

To our knowledge, no previous study has simultaneously examined the combined effects of psychological, social, and physical factors on fall risk perception in this specific population. Understanding the interplay and relative weight of these modifiable determinants is clinically essential, as it would enable healthcare providers to design targeted, multi-component interventions that accurately calibrate patients’ risk awareness, promote proactive preventive behaviours, and ultimately reduce fall-related rehospitalisations and poor functional outcomes in this vulnerable group. Therefore, the present study investigated the current status and multidimensional influencing factors of falls risk perception in elderly patients after hip fracture surgery, with the goal of providing an empirical basis for developing individualized fall prevention strategies that can be feasibly integrated into routine postoperative nursing care.

## Methods

### Participants

A convenience sampling method was used in this study. Elderly patients admitted to the Department of Orthopaedics of Mianyang Central Hospital from December 2024 to November 2025 after hip fracture surgery were selected as the study population. Inclusion criteria: (1) Hip fracture confirmed by imaging examination (MRI, X-ray, CT); (2) Fresh fracture with time from fracture ≤ 7 days; (3) Age ≥ 60 years; (4) Unilateral hip fracture; (5) All patients underwent surgical treatment for hip fracture and it was their first hip fracture surgery; (6) Ability to bear weight and walk normally before the fracture. Exclusion criteria: (1) Presence of liver or kidney dysfunction, multiple organ injury, etc.; (2) Psychiatric illness or cognitive impairment; (3) Concurrent malignant tumour or systemic infection; (4) Presence of muscle weakness, extreme debility, or other diseases that prevent the patient from getting out of bed after surgery.All methods were performed according to the relevant guidelines and by the Declaration of Helsinki. Biomedical ethics committee of Mianyang Central Hospital approved the study (S20250383-01). All participants gave informed voluntary consent following assurance of the confidentiality of the study questionnaires.

Referring to relevant literature, the required sample size for a survey study is 5–10 times the number of items. The general information questionnaire contained 21 items, the Fall Risk Perception Questionnaire had 3 dimensions, the Perceived Social Support Scale had 3 dimensions, and the anxiety-depression scale had 2 dimensions, giving a total of 29 dimensions/items. Thus the required sample size was 29 × (5–10) = 145–290 cases. Assuming a 10% invalid questionnaire rate, the sample size should be 162–323 cases. The questionnaire system(Sojump) required complete responses to all items, and submissions with a completion time of less than 3 min were automatically rejected. After data collection, questionnaires were independently reviewed by two researchers; those with all items answered using the same option were identified as invalid and excluded. A total of 170 questionnaires were returned, after excluding 8 invalid responses, 162 valid questionnaires were finally included in the analysis.

### Research instruments

#### General information questionnaire

A self-designed questionnaire was developed by the researchers based on the study purpose and after reviewing relevant literature. It included items on age, sex, education level, occupation, body mass index (BMI), place of residence, living arrangement, method of payment for medical expenses, marital status, and history of falls in the past year.

#### Fall Risk Perception Questionnaire for patients (FRPQ)

This scale was originally developed by CHOI [19] and subsequently adapted into Chinese by Bao et al. [15]. The Chinese version of the FRPQ consists of three dimensions (environmental factors, 13 items; personal activity factors, 8 items; physical condition factors, 5 items), 26 items in total. It uses a Likert 4-point scale, with total scores ranging from 0 to 78. Each item is scored from 0 (“no risk at all”) to 3 (“extremely high risk”). Higher total scores indicate a greater perceived risk of falling. The scale-level content validity index (S-CVI) based on universal agreement was 0.867, the average S-CVI was 0.909, and the item-level content validity index (I-CVI) ranged from 0.860 to 1.000; additionally, the item critical ratios (CR) ranged from 9.020 to 18.373, the Cronbach’s αwas 0.942, and the split-half reliability was 0.808.

#### Perceived Social Support Scale (PSSS)

The PSSS was originally developed by Blumenthal et al. [20] in 1987 and later translated and modified into Chinese by Jiang Qianjin. The Chinese version of the PSSS has 12 items grouped into three subscales: family support, friend support, and other support. It measures the extent to which an individual feels supported by family, friends, and other people, and the total score reflects the individual’s overall perceived social support. The scale uses a 7-point scoring method (1 to 7), with a total score ranging from 12 to 84. Scores of 12–36 indicate low support, 37–60 moderate support, and 61–84 high support. The PSSS has good reliability and validity, with an internal consistency coefficient of 0.93 for the total scale, and subscale internal consistency reliabilities of 0.83 for family support, 0.82 for friend support, and 0.76 for other support, all meeting psychometric standards.

#### Anxiety and depression scale

This scale was developed by W.K. Zung and is one of the scales recommended by the U.S. Department of Health, Education, and Welfare for psychopharmacological research. Because it is easy to use and can intuitively reflect patients’ subjective feelings of depression and their changes during treatment, it has been widely used for initial screening of outpatients, mood state assessment, surveys and research [21]. The simplified anxiety subscale and simplified depression subscale were used to assess depressive symptoms for outpatients and inpatients. The Cronbach’s α coefficients were 0.839 and 0.884, and the test-retest reliabilities were 0.846 and 0.635, respectively. Clinical scoring criteria defined scores of < 4 on the simplified anxiety subscale and < 3 on the simplified depression subscale as indicating no depressive symptoms. The simplified anxiety subscale consists of 7 items, using a Likert 4 point scale, with a total score of 21; higher scores indicate higher levels of anxiety. The simplified depression subscale has 9 items, also using a Likert 4 point scale, with a total score of 27; higher scores indicate higher levels of depression.

#### Chinese version of the international fall efficacy scale [22]

This scale is used to measure an individual’s fear of falling. It consists of 16 items, each scored on a Likert 4 point scale from 1 to 4. The total score ranges from 16 to 64, with higher scores indicating greater fear of falling. The scale has been validated in patients with Parkinson’s disease, spinal cord sclerosis, and hip fracture, and has shown good reliability, validity, and psychometric properties. The Cronbach’s α coefficient of this scale was 0.921, the split-half reliability coefficient was 0.914, and the test-retest reliability coefficient was 0.906.

#### Pain

Pain levels were assessed using a 10‑cm Visual Analogue Scale (VAS), with scores ranging from 0 to 10. Pain was evaluated at two time points: on the day of surgery and at first postoperative ambulation. For analysis, pain scores were categorised as none (0), mild (1–3), moderate to severe (4–10), consistent with standard clinical practice.

### Data collection

Before formal data collection, permission was obtained from the orthopaedic ward management. The researchers strictly screened the participants according to the inclusion and exclusion criteria, and patients who were able to get out of bed after surgery were selected to receive the questionnaire. Before distributing the questionnaires, a standardised instruction was used to explain the purpose and significance of the study to the participants. After obtaining informed consent from the patients, the questionnaires were distributed and collected on the spot. Questionnaires with three or more missing items or with regular/patterned responses were considered invalid. A total of 162 questionnaires were distributed, none of which were invalid, resulting in 162 valid questionnaires and an effective response rate of 100%.

### Statistical methods

All data were entered and verified by two individuals. Statistical analysis was performed using SPSS version 24.0. Continuous variables following a normal distribution were expressed as mean±standard deviation (x̄±s). Comparisons between two groups were made using the t-test, and comparisons among multiple groups using one-way analysis of variance (ANOVA). Categorical variables were presented as frequencies (percentages). Pearson correlation analysis was used to examine correlations between variables. Categorical data were compared using the chi-square (χ²) test. Multiple linear regression analysis was used to identify factors influencing falls risk perception after hip fracture surgery in elderly patients. A P-value < 0.05 was considered statistically significant.

## Results

### Falls risk perception, anxiety, depression and perceived social support in elderly orthopaedic patients after hip fracture surgery

Among the 162 elderly patients included in this study, the mean age was 78.33 ± 9.32 years. The total score of falls risk perception in elderly patients after hip fracture surgery was at a moderate to low level (35.43 ± 12.00). Among the dimensions, the personal activity factor scored the highest and the physical condition factor scored the lowest. The scores for anxiety, depression and perceived social support are shown in Table 1.

**Table 1:** Scores of falls risk perception dimensions, anxiety, depression, and perceived social support dimensions in elderly orthopaedic patients after hip fracture surgery (*n* = 162)

Item	Number of items	Score range	Mean score (x̄±s)
Falls risk perception	26	0 ~ 78	35.43±12.00
Environmental factors	13	0 ~ 39	14.86±5.01
Personal activity factors	8	0 ~ 24	15.06±5.50
Physical condition factors	5	0 ~ 15	5.52±4.14
Anxiety	7	0 ~ 21	2.37±3.20
Depression	9	0 ~ 27	5.77±4.58
Perceived social support	12	0 ~ 84	68.46±10.10
Family support	4	0 ~ 28	26.18±2.06
Friend support	4	0 ~ 28	16.09±7.87
Other support	4	0 ~ 28	26.19±2.19

### Univariate analysis of falls risk perception after hip fracture surgery in elderly orthopaedic patients

The total score of falls risk perception was used as the dependent variable, and the independent variables included age, sex, marital status, education level, payment method, occupation, pain level on the day of surgery, and pain level at first postoperative ambulation. Univariate analysis showed that age, education level, payment method, and pain level at first postoperative ambulation were significantly associated with falls risk perception (*P* < 0.05). Patients with moderate‑to‑severe pain had significantly lower falls risk perception scores than those without pain, shown in Table 2.

**Table 2:** Univariate analysis of falls risk perception after hip fracture surgery in elderly orthopaedic patients (*n* = 162)

Item	Category	number(*n*,%)	Falls risk perception (‾x ± s)	t/F	*P*
Age (years)	60 ~ 69	27(16.7)	28.26±11.64	13.30	< 0.001
	70 ~ 79	60(37.0)	32.95±10.88		
	> 80	75(46.3)	40.00±11.25		
Gender	Male	48	33.58±12.72	1.625	0.204
	Female	114	36.21±11.66		
Education level	Primary school or below	96(59.3)	37.94±11.25	3.782	0.012
	Junior high school	32(19.8)	30.81±13.71		
	High school	21(13.0)	33.24±11.67		
	College or above	13(8.0)	31.85±9.36		
Current place of residence	Rural	59	35.43 ± 11.95	1.842	0.177
	Urban	103	34.47 ± 11.97		
Current living arrangement	Living with children	115	36.26 ± 12.76	0.945	0.391
	Living alone / other	33	33.42 ± 9.87		
	Living with spouse and children	14	33.36 ± 9.71		
Occupation	Unemployed	29	38.14 ± 9.11	1.510	0.224
	Retired	71	33.77 ± 12.05		
	Farmer	62	36.06 ± 12.97		
Payment method	Employee basic medical insurance	60	34.77 ± 12.60	3.094	0.048
	Resident basic medical insurance	91	36.81 ± 11.54		
	Other	11	27.64 ± 9.73		
Monthly household income (RMB)	< 5000	103	36.81 ± 12.12	2.491	0.086
	5000–10,000	47	33.89 ± 11.57		
	> 10,000	12	29.67 ± 10.97		
Pain level on the day of surgery	Mild pain	118	35.96 ± 12.29	1.953	0.145
	Moderate‑to‑severe pain	16	38.19 ± 11.27		
	No pain	28	31.64 ± 10.59		
Pain level at first postoperative ambulation	Mild pain	134	35.43 ± 11.36	7.263	< 0.001
	Moderate‑to‑severe pain	10	29.22 ± 12.31		
	No pain	18	38.43 ± 11.36		

### Correlation analysis of falls risk perception with anxiety, depression, and perceived social support in elderly orthopaedic patients after hip fracture surgery

The correlation analysis showed that falls risk perception was negatively correlated with anxiety score (*r*=-0.358) and depression score (*r*=-0.520) (*P* < 0.01), and positively correlated with perceived social support (*r* = 0.444) and fall efficacy (*r* = 0.600) (*P* < 0.01), shown in Table 3.

**Table 3:** Correlation analysis between falls risk perception and other variables

Variable	Falls risk perception	Anxiety	Depression	Perceived social support	Fall efficacy
Falls risk perception	1				
Anxiety	-0.358**	1			
Depression	-0.520**	0.525**	1		
Perceived social support	0.444**	-0.210*	-0.470**	1	
Fall efficacy	0.600**	-0.358**	-0.520**	0.444**	1

*P < 0.05, **P < 0.001

### Multiple linear regression analysis of factors influencing falls risk perception in elderly patients with hip fracture

Independent variables with *P* < 0.05 in univariate analysis were entered into the multiple linear regression model. In addition, based on theoretical frameworks ( the Health Belief Model), empirical evidence from previous literature, and clinical relevance, we also forced the inclusion of several theoretically important variables regardless of their univariate significance, to adjust for potential confounding effects. age, payment method, education level, pain level at first postoperative ambulation, perceived social support, anxiety, depression, and fall efficacy were included as independent variables in a multiple stepwise regression analysis to establish a regression equation. According to the requirements of multiple stepwise regression analysis for independent variables, multi‑categorical independent variables were dummy‑coded before statistical analysis and then entered into the regression model. The assignment of each independent variable and the dummy variable settings are shown in Table 4.

**Table 4:** Variable assignment and coding of general characteristics in elderly patients with hip fracture

Independent variable	Assignment
Age(years)	60–69=(0, 0, 0)
	70–79=(0, 1, 0)
	> 80=(0, 0, 1)
Payment method	Employee basic medical insurance=(0, 0, 0)
	Resident basic medical insurance=(0, 1, 0)
	Other=(0, 0, 1)
Education level	Primary school or below=(0, 0, 0, 0),
	Junior high school=(0, 1, 0, 0)
	High school=(0, 0, 1, 0)
	College or above=(0, 0, 0, 1)
Pain level at first postoperative ambulation	no pain=(0, 0, 0)
	Mild pain=(0, 1, 0)
	Moderate‑to‑severe pain=(0, 0, 1)
Perceived social support	Raw value
Anxiety	Raw value
Depression	Raw value
Fall efficacy	Raw value

Falls risk perception was a continuous variable, and the residuals were approximately normally distributed. The Durbin‑Watson test value in this study was 1.736, indicating that the samples were independent of each other. The collinearity diagnostics showed that tolerance (TOL) values ranged from 0.58 to 0.81, all greater than 0.10; the variance inflation factor (VIF) values ranged from 1.23 to 1.61. It is generally considered that when VIF is less than 5, there is no significant multicollinearity among the variables. Therefore, it can be concluded that the independent variables in this study were independent of each other and had no multicollinearity.

The results showed that six variables entered the regression equation as independent factors influencing falls risk perception in elderly patients with hip fracture: fall efficacy, perceived social support, anxiety, depression, education level, and pain level at first ambulation. The regression coefficients indicated that patients with higher scores of fall efficacy and perceived social support had higher levels of falls risk perception. Compared with patients with primary school education or below, those with high school or technical secondary school education had higher levels of falls risk perception. Higher scores of anxiety and depression were associated with lower levels of falls risk perception. Compared with patients who had no pain at first postoperative ambulation, those with moderate‑to‑severe pain had lower levels of falls risk perception. The six factors together explained 39.5% of the variance in the total falls risk perception score (adjusted R² = 0.395), shown in Table 5.

**Table 5:** Regression analysis of factors influencing falls risk perception after hip fracture surgery in elderly orthopaedic patients

Variable	B	Stand error	Standardized β	t	*P*	VIF
Constant	2.154	0.370	-	5.82	< 0.001	-
Fall efficacy	0.248	0.071	0.302	3.47	0.001	1.61
Perceived social support score	0.163	0.058	0.215	2.79	0.006	1.35
Anxiety score	-0.291	0.096	-0.258	-3.02	0.003	1.45
Depression score	-0.205	0.085	-0.189	-2.41	0.017	1.72
High school(reference: primary school or below)	0.387	0.177	0.146	2.18	0.031	1.23
Moderate-to-severe pain at first ambulation (reference: no pain)	-0.513	0.191	-0.224	-2.68	0.008	1.27

R2 = 0.428, adjusted R2 = 0.395; F = 12.86,*p* < 0.001

## Discussion

### Current status of falls risk perception after hip fracture surgery in elderly orthopaedic patients

Our study showed that the total score of falls risk perception after hip fracture surgery in elderly patients was 35.43 ± 12.00, which was at a moderate‑to‑low level compared with the theoretical midpoint of 52 on the scale. At the same time, this result was lower than that reported by Chen et al. [23] in elderly hospitalised patients with cancer. The reason may be related to the type of disease: hip fracture has a smaller psychological impact on patients than cancer, and the cost of surgical treatment for hip fracture is relatively lower with a shorter treatment period, resulting in lighter psychological and economic burdens on patients and their families. Patients are prone to the illusion of “safety after surgery”, underestimate fall‑related risks, overestimate their own abilities and engage in inappropriate personal activities, thus leading to adverse outcomes [14, 24–26]. At present, many international studies have shown that patients are unable to accurately assess their own fall risk [12]. In addition, there were differences between different dimensions of falls risk perception. In this study, the personal activity factor had the highest scoring rate (62.7%) and the physical condition factor the lowest (36.8%), consistent with the findings of a related study [27], reflecting that elderly patients are relatively sensitive to risks in activity situations but poorly assess their own physical condition. Hughes et al. [14] found that older adults with better self‑rated health had lower falls risk perception, which indirectly suggests that professional guidance is needed to help elderly patients objectively understand their own abilities. Therefore, it is recommended that clinical staff pay attention to fall risk assessment in this elderly population, strengthen risk education regarding changes in patients’ physical function, and guide them to rebuild a comprehensive and rational perception of fall risk.

### Analysis of factors influencing falls risk perception after hip fracture surgery in elderly orthopaedic patients

The six independent predictors identified in our final model were selected based on the Health Belief Model (HBM) [28], which posits that health related risk perception is influenced by perceived susceptibility, self‑efficacy, demographic variables, psychosocial cues, and structural barriers. Drawing on this framework, we conceptualised fall risk perception as a cognitive outcome shaped by internal emotional states, self‑evaluative beliefs, acute somatic experience, and external resources. Importantly, these factors do not operate in isolation: anxiety, depression, and pain may reciprocally reinforce each other to distort risk perception, whereas social support and higher education may buffer these negative effects and promote more accurate risk appraisal.

### Fall efficacy

Fall efficacy was a positive predictor of falls risk perception (β = 0.302, *P* = 0.001), consistent with Chen et al. [27]., who reported a similar positive correlation between aging attitudes and risk perception. Although seemingly counterintuitive, this reflects the double‑edged sword effect of fall efficacy: individuals with low efficacy tend to avoid activities due to excessive fear, lacking experience and discernment of real risks, leading to blunted risk perception; conversely, those with high efficacy, based on successful experiences or systematic training, can more accurately identify dangerous actions and environments, forming more objective risk judgement. Clinically, interventions should not simply aim to “reduce fear of falling” but, through graded activity training and feedback on successful experiences, help patients establish self‑efficacy matched to their abilities, neither overly anxious nor blindly overconfident.

### Pain level before first ambulation

Patients with moderate-to-severe pain at first postoperative ambulation had lower falls risk perception in both univariate and regression analyses. Severe pain may occupy cognitive resources, reducing attention to environmental risks, or shift focus towards completing ambulation quickly, thereby underestimating fall possibility. Pain is also directly proportional to fear of falling [29]., and as Slovic proposed in the psychometric paradigm, fear is a fundamental element of risk perception [14, 30], potentially leading to avoidance decisions and pessimistic risk appraisal [31]. Consistent with our findings, previous studies have reported that inadequate pain control impairs patients’ awareness of environmental hazards during early mobilisation [32].Therefore, healthcare professionals should perform pain assessment and management before first ambulation [33], using analgesics to improve comfort, reduce fear, and prevent excessively low risk perception [34]. Optimising perioperative pain management through multi‑modal strategies—preventive analgesia, customised dosing, and graded treatment—is particularly crucial [1].

### Anxiety and depression

Contrary to the common intuition that “anxiety and depression amplify risk,” we found that patients with higher anxiety/depression levels perceived lower fall risk, consistent with Chen et al. [27]. Negative emotions may induce risk numbness(diminished sensitivity to fall threats after repeated exposure, leading to underestimation of risk) or learned helplessness (a passive psychological state in which patients believe their actions cannot alter outcomes, reducing motivation for prevention), weakening alertness to falls. Depressed patients have reduced cognitive function, especially slower processing speed, suggesting that depression affects perceptual judgement through reduced cognitive resources [14], biasing falls risk perception [35]. Anxiety and depression can also be exacerbated by pain, creating a vicious cycle that impairs rehabilitation exercise [17]. This suggests that fall prevention should consider the interaction of depression, cognitive function, and physical pain; for patients who respond poorly to fall interventions, simultaneous assessment of emotional status, cognitive function, and pain is recommended [12].

### Perceived social support level

Perceived social support was a positive predictor of falls risk perception: higher support was associated with higher risk perception. In this study, support levels were relatively high (scoring rate 81.5%), with family support and other support scoring highest (both 93.5%), while friend support scored 57%. Elderly patients can obtain material, informational and emotional support from multiple sources, which can improve their perceived risk [36] and enable more accurate self-assessment. Our positive correlation between social support and risk perception is consistent with findings by Trevisan et al. [37] in postoperative elderly populations, though it contrasts with some studies in community‑dwelling older adults where social support was associated with lower fear of falling. This discrepancy may reflect the different constructs assessed (risk perception vs. fear of falling) and the acute postoperative context.

Good social support also alleviates worry, tension, and anxiety [38]. in this study, support was negatively correlated with depression (*r* = − 0.461, *P* < 0.001), indicating that higher perceived support may both alleviate depressive symptoms and facilitate more accurate risk appraisal. Clinically, healthcare workers should maintain active communication, provide timely health education and scientific guidance, and offer assistive devices (canes, walkers, wheelchairs). Improving infrastructure accessibility and promoting device use can reduce worry about falling, increase activity range and social integration, and contribute to healthy ageing [14].

### Education level

Using primary education or below as the reference, patients with high school or technical secondary education had significantly higher falls risk perception scores (β = 0.387, *P* = 0.031). One possible explanation is that education may be associated with health literacy and information processing ability: more educated patients may better understand fall risk education materials and translate abstract knowledge into concrete situational judgements, thereby achieving more accurate risk appraisal. This finding is consistent with studies by Thitima et al. [39], who reported that higher health literacy is associated with more accurate risk perception in older adults, though it contrasts with studies suggesting education is unrelated to fear of falling—a related but conceptually distinct construct. No significant difference was found for the college degree or above group, possibly due to the small sample size; therefore, further validation with a larger cohort is needed. For patients with low education, more intuitive educational methods (e.g., illustrated materials, bedside simulation demonstrations, short videos) should be considered, alongside family caregiver education to compensate for limited health literacy.

### Falls history

Although previous studies have shown that a history of falls is associated with higher fall risk perception in older adults [40], our study did not find fall history to be a significant predictor in the multivariate model. This discrepancy may be explained by differences in study populations (community-dwelling vs. postoperative inpatients), the time interval between the last fall and the survey, or the possibility that the acute postoperative experience (pain, mobility restriction, and hospital environment) may temporarily attenuate the influence of prior fall history on current risk perception. Future longitudinal studies are warranted to explore this relationship over the recovery trajectory.

### Limitations

This study has several limitations. First, the sample was recruited from a single centre, which may limit the generalisability of the findings. Second, the cross‑sectional design of this study precludes any causal inferences regarding the relationships between the identified predictors and falls risk perception. Therefore, the associations reported here should be interpreted as correlational rather than causal. Third, objective measures such as prior fall history and balance function were not included in the analysis. Fourth, the sample size of the college degree or above subgroup was relatively small, which may have affected the stability of subgroup comparisons. Future multicentre and longitudinal studies are needed, incorporating objective risk assessment tools, to further explore the cognitive mechanisms underlying falls risk perception.

## Conclusions

The overall level of falls risk perception after hip fracture surgery in elderly patients is moderate-to-low. Age, pain level at first ambulation, anxiety, depression, perceived social support, fall efficacy and education level are independent influencing factors. Clinically, particular attention should be paid to older patients, those with low education level, those with mood disorders, and those with significant postoperative pain. Comprehensive interventions including adequate analgesia, psychological counselling, mobilisation of social support, and efficacy-enhancing training may help patients establish accurate and moderate awareness of fall risk, thereby enabling them to take proactive preventive measures and reduce the incidence of falls.

## Data Availability

The datasets used and analysed during the current study are available from the corresponding author upon reasonable request. All data and relevant materials are stored at Mianyang Central Hospital, No. 12 Changjiaxang, Fucheng District, Mianyang, Sichuan, China.
